# How to Monitor Cardiac Complications of Immune Checkpoint Inhibitor Therapy

**DOI:** 10.3389/fphar.2020.00972

**Published:** 2020-06-26

**Authors:** Paolo Spallarossa, Matteo Sarocchi, Giacomo Tini, Eleonora Arboscello, Matteo Toma, Pietro Ameri, Italo Porto

**Affiliations:** ^1^ Cardiovascular Disease Unit, IRCCS San Martino Policlinic Hospital–IRCCS Italian Cardiovascular Network, Genova, Italy; ^2^ Department of Internal Medicine, University of Genova, Genova, Italy; ^3^ Department of Emergency, IRCCS San Martino Policlinic Hospital, University of Genova, Genova, Italy

**Keywords:** checkpoint inhibition therapy, myocarditis, cardiotoxicity, screening, cardiovascular side effects, troponin

## Abstract

Immune-checkpoint inhibitors (ICIs) represent a successful paradigm in the treatment of cancer. ICIs elicit an immune response directed against cancer cells, by targeting the *so-called* immune checkpoints, key regulators of the immune system that when stimulated can dampen the immune response to an immunologic stimulus. Such response, however, is not entirely tumor-specific and may result in immune-related adverse events (irAEs), involving a number of organs and systems. Cardiovascular (CV) irAEs are rare, although potentially severe. In particular, several cases of ICI-related myocarditis with life-threatening course have been reported: the possibility of fulminant cases, thus, requires a high level of awareness among both oncologists and cardiologists. Aggressive work-up and management of symptomatic patients taking ICIs is fundamental for early recognition and initiation of specific immunosuppressive therapies. Notably, myocarditis occurs within few weeks from ICIs initiation, offering opportunity for a targeted screening. Troponin testing is the cornerstone of this screening, yet uncertainties remain regarding timing and candidates. Moreover, troponins positivity should be carefully interpreted. We herein review the main aspects of ICI-related myocarditis and suggest a practical approach. In particular, we focus on the opportunities that a baseline CV evaluation offers for subsequent management by collecting clinical and instrumental data, essential for the interpretation of troponin results, for differential diagnosis and for the formulation of a diagnostic and therapeutic workup.

## Introduction****

The clinical introduction of immune checkpoint inhibitors (ICIs) has resulted in impressive results in the treatment of some advanced and previously minimally responsive cancers (Farkona et al., 2016). These drugs are being increasingly used for a large number of solid and hematological malignancies, also at early stages (Moslehi et al., 2018). ICIs target an integrated pathway of membrane inhibitory and stimulatory receptors (the *so-called* immune checkpoints), crucial for regulating T-cell activation and preventing autoimmunity and excessive inflammatory response (Spallarossa et al., 2018). Cancer exploits these pathways to escape from immune surveillance. Many tumor cells over-express a ligand, the programmed death-ligand 1 (PD-L1), which binds inhibitory receptors expressed on T lymphocytes, such as cytotoxic T lymphocyte-associated protein-4 (CTLA-4) and programmed death protein-1 (PD-1). The main effect of ICIs, which include antibodies against CTLA4 (ipilimumab) (Eggermont et al., 2015), PD-1 (nivolumab, pembrolizumab, cemiplimab) (Herbst et al., 2016; Ansell, 2017; Guo et al., 2017; Herbaux et al., 2017) and PD-L1 (atezolizumab, avelumab, durvalumab) (Massard et al., 2016; Apolo et al., 2017; Peters et al., 2017), is to impede these interactions and enhance a T-cell reaction against tumors. In this way, ICIs elicit an immune response primarily directed against cancer cells. However, such response is not entirely tumor-specific and may enhance the development of immune-related adverse events (irAEs), involving a number of organs and systems.

irAEs vary among individual patients, but frequently include colitis, dermatitis, hepatitis, pneumonitis, colitis, and endocrinopathies (Eigentler et al., 2016; Davies and Duffield, 2017; De Velasco et al., 2017; Genova et al., 2017; Lee et al., 2019). Cardiovascular (CV) irAEs are rarer, but carry significant mortality and morbidity. They range from myocarditis to pericarditis, tako-tsubo syndrome, acute coronary syndrome (ACS), and vasculitis (Hu et al., 2019).

Myocarditis has drawn most attention, for two reasons. Firstly, ICI-related myocarditis has an ominous prognosis, since it may result in life-threatening heart failure or arrhythmias, sometimes with a fulminant course (Brahmer et al., 2018; Spallarossa et al., 2019). Secondly, the risk of ICI-related myocarditis requires specific clinical surveillance, unlike other CV irAEs.

In the present work, we will focus on ICI-related myocarditis, with the aim of better defining the strategies for early recognition of this possibly serious irAE.

### Why Monitoring Patients Treated With Immune Checkpoint Inhibitor

Though only few cases of severe myocarditis were reported in clinical trials of ICIs, subsequent observations suggest a significantly higher incidence (Spallarossa et al., 2018; Moslehi et al., 2018; Spallarossa et al., 2019), from 0.04% up to 1.14% (Palaskas et al., 2020), depicting ICI-related myocarditis as a severe form of cardiotoxicity with a fulminant clinical course often resulting in cardiogenic shock, advanced conduction disturbances, and ventricular arrhythmias. However, as most of the available evidence derives from case reports, case series, or retrospective studies (Johnson et al., 2016; Berg et al., 2017; Martinez-Calle et al., 2018; Moslehi et al., 2018; Samara et al., 2018; Yamaguchi et al., 2018), it may be flawed by reporting bias (i.e., only the most severe cases were described), whereas asymptomatic or mildly symptomatic forms may have gone unidentified or unreported.

Thus, as the incidence of ICI-related myocarditis with a subtle presentation is still unknown, as well as its course if left untreated (Sarocchi et al., 2018), it cannot be excluded that in some cases subclinical myocarditis due to ICIs may even be self-limiting (Spallarossa et al., 2019). Hence, CV monitoring of patients receiving ICI therapy should not merely sought to diagnose overt cases of myocarditis; rather, its aim should be to recognize subclinical or asymptomatic cases, in order to avoid their progression.

### How to Manage Immune Checkpoint Inhibitor-Related Myocarditis

The management of ICI-related myocarditis represents a clinical challenge. We herein propose a five steps approach for the management of ICI-related myocarditis. Of these five points, treatment only represents the final one.

#### Step 1: Awareness

Cardio-Oncology is a field where interaction between specialties is of paramount importance (Tini and Spallarossa, 2019). This concept holds true for the management of patients receiving ICIs (Veronese and Ammirati, 2019). Even in centres where a cardio-oncology programme is not structured, it is advisable to organise an integrated team taking care of patients treated with ICIs. Briefly, this collaboration requires:

1. Oncologist awareness: the oncologist should not only acknowledge the possibility that ICIs may result in CV irAEs, but also be familiar with the fundamentals of myocarditis (from symptoms to differential diagnosis). In particular, given the possibility of fatal or rapidly progressing ICI-related myocarditis (Johnson et al., 2016), the oncologist should be aware that any myocarditis process due to ICI therapy may represent a clinical emergency.
2. Cardiologist awareness: a referring cardio-oncologist or cardiologist should be involved in the evaluation and management of patients receiving ICIs. The general cardiologist, especially if a cardio-oncology programme is not present, may not be familiar with ICI-related myocarditis.
3. Involvement of other specialists: severe cases of ICI-related myocarditis are anecdotally associated with neuro-muscular irAEs (Behling et al., 2017; Fazel and Jedlowski, 2019; Moreira et al., 2019), and the involvement of a neurologist in the evaluation of cases where signs or symptoms of myositis are present may be warranted. Moreover, CV irAEs may be associated to irAEs in other districts (Puzanov et al., 2017) and may require multidisciplinary evaluation with endocrinologists, pneumologists, dermatologists, and gastroenterologists. On the other hand, any health care professional caring for patients receiving ICIs should be aware and pay attention to occurrence of cardiac symptoms, especially when irAEs not involving the heart are the initial reason for medical attention.
4. Patient education: patients scheduled to receive ICIs should be educated to recognize cardiac symptoms potentially caused by myocarditis.
5. Immunotherapy “patient wallet card”: all patients should be given a summary intended to be shown to any health care provider including the primary care physician—when needed —or in the emergency room (i.e., https://www.ons.org/clinical-practice-resources/immunotherapy-patient-wallet-card). This card should include information about:
  - the referring oncologist and how to contact her/him;
  - the cancer treated with ICIs;
  - the potential side effects of ICIs, with emphasis to myocarditis;
  - how initially manage specific ICIs side effects.
6. Standard procedures: screening programmes, exams, and management strategies for patients diagnosed with ICI-related myocarditis should be established by a multidisciplinary team.

#### Step 2: Baseline Cardiology Evaluation

To date, there is no evidence that a pre-existing cardiac condition identifies patients at higher risk for ICI-related myocarditis (Spallarossa et al., 2018; Tocchetti et al., 2018). We believe, however, that routine baseline cardiology evaluation of all patients scheduled to receive ICIs is both feasible and reasonable. As discussed in the following paragraphs, presenting symptoms, signs, and laboratory data of ICI-related myocarditis can be mild and/or unspecific, and might be attributed to a broad spectrum of CV diseases, including acute and chronic coronary syndromes, left ventricular dysfunction, and arrhythmias. Collecting baseline clinical, ECG and echocardiography data would allow to recognize any change occurring during ICI therapy, facilitating early diagnosis of ICI-related myocarditis *versus* other cardiac disorders (Bonaca et al., 2019; Spallarossa et al., 2019).

#### Step 3: Screening Strategies

Whether is appropriate to screen for ICI-related myocarditis remains unknown. To be effective, such a strategy should result in early detection of myocarditis (and other cardiac irAEs), prompting an appropriate management plan to prevent a potentially severe course (Spallarossa et al., 2019). Nevertheless, screening should also be practically feasible and—most importantly—should not result in inappropriate withholding of a life-saving therapy.

In asymptomatic patients, measurement of cardiac troponins is the strategy most often recommended to screen for ICI-related myocarditis, despite this approach having inherent limitations (Spallarossa et al., 2019). ICI-related myocarditis usually occurs early during treatment, and is more common with combination therapy, typically the anti-CTLA4 molecule ipilimumab with an inhibitor of PD-1/PD-L1 interaction (mostly pembrolizumab or nivolumab) (Mahmood et al., 2018; Ball et al., 2019; Fan et al., 2019). Screening is thus usually advised in the first 12 weeks of treatment, whereas it is not clear whether it should be performed in the case of single ICI therapy or only with combinations (Puzanov et al., 2017; Brahmer et al., 2018). Since myocarditis may be associated with myositis, it may be reasonable to additionally assess creatine phosphokinase (CPK) values (Brahmer et al., 2018). In our institution, we adopt a screening strategy with troponin testing in all type of ICIs therapies, and associate CPK measurement to increase specificity. Indeed, a myocardial injury (i.e., ACS) associated with a trivial increment of troponin values does not usually cause the raise of other less sensitive markers of myocardial damage, such as CPK.

**Figure 1:**
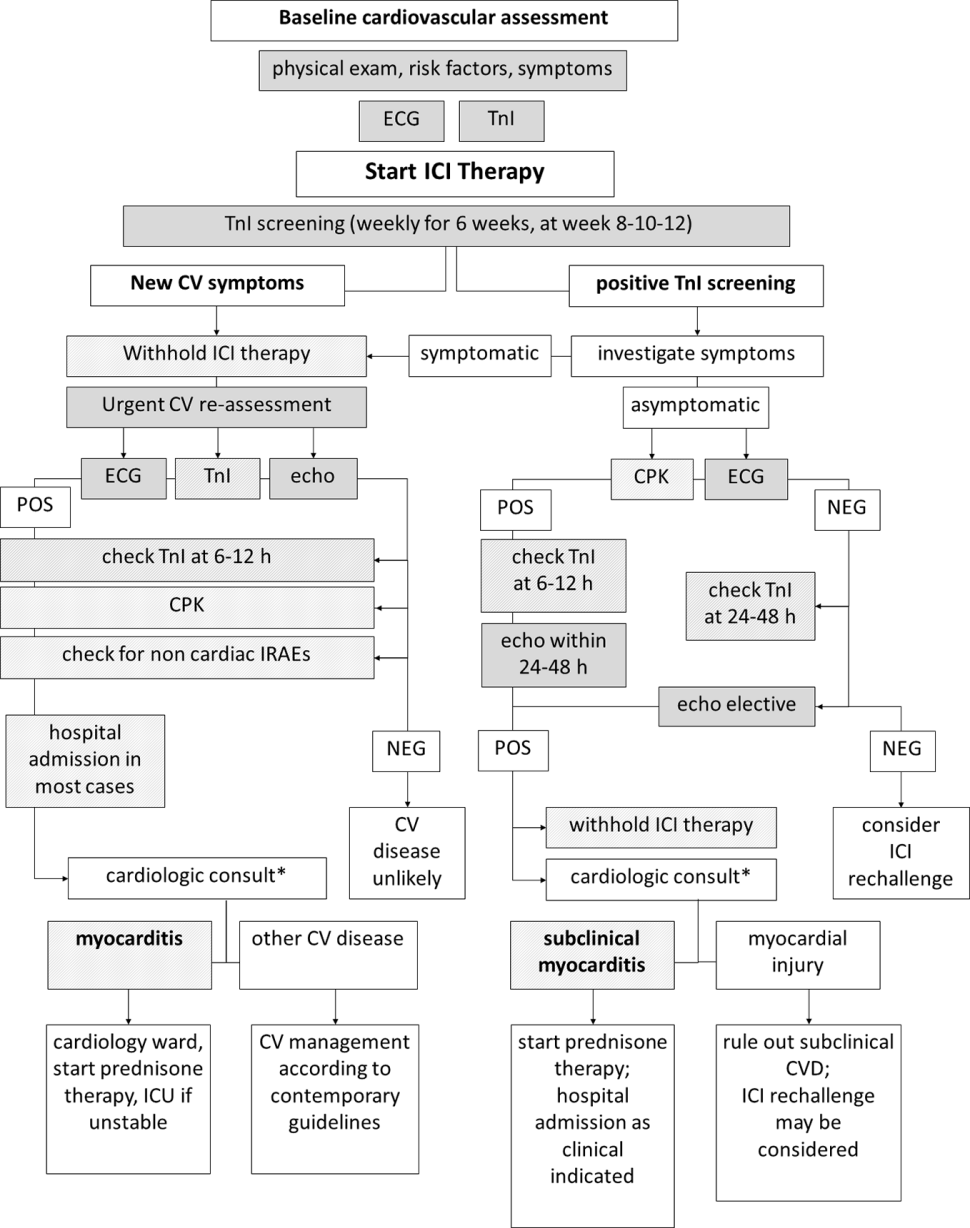
Diagnostic workup of immune-checkpoint inhibitor (ICI)-related myocarditis. Diagnostic workup of ICI-related myocarditis may begin in the setting of troponin screening (*right*) or due to onset of symptoms during ICI therapy (*left*). In both cases, collecting data from multiple sequential clinical and instrumental steps is mandatory to reach a diagnosis. Note that timing of repeated troponin testing differs due to presence/absence of symptoms. *Differential diagnosis result—at a larger extent—from the integration and interpretation of clinical, anamnestic, laboratory, ECG, and echocardiography data, as highlighted in **Figure 2**.

**Figure 2:**
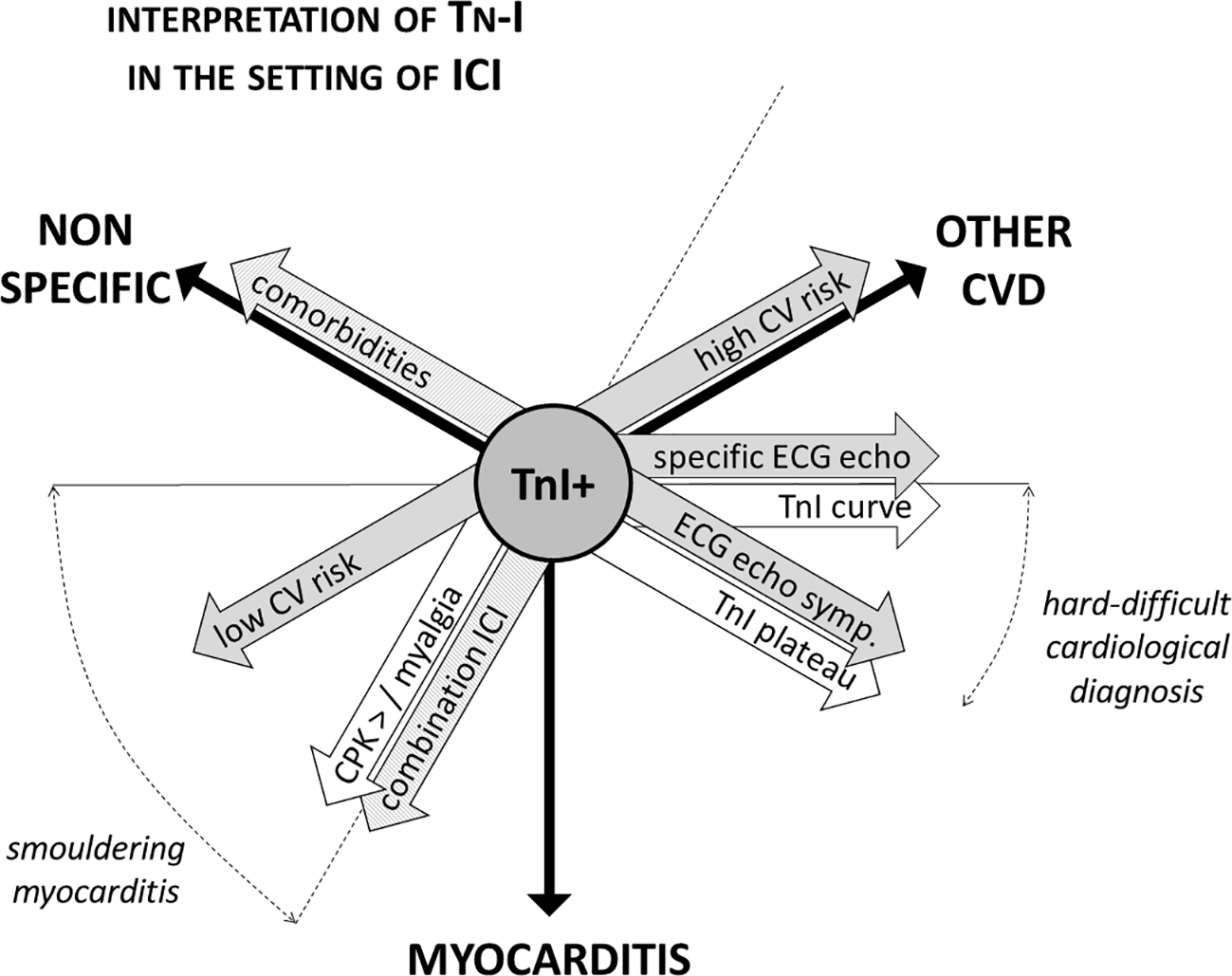
Interpretation of troponin positivity in the setting of ICI therapy. Troponins positivity in patients on ICI therapy is not specific of myocarditis, and troponin testing always needs to be carefully interpreted in the light of a comprehensive clinical evaluation. The aspects to be considered are manifold: pharmacological (single or combination ICI therapy, presence of other irAEs), clinical (CV risk profile, presence of comorbidities and/or of stressors as anemia), instrumental (ECG and echocardiographic abnormalities), and biochemical [dynamics of troponin elevation, concomitant rising of creatine phosphokinase (CPK)]. For instance, ECG and echocardiographic focal abnormalities (i.e., segmental, corresponding to coronary territory distribution) are more common in the setting of coronary artery disease. Troponins elevation follows a typical dynamic curve in the setting of acute coronary syndromes, while frequently show a steady increase in myocarditis. ICI, immune checkpoints inhibitors; CV, cardiovascular; irAE, immune-related adverse events; CVD, cardiovascular disease; TnI, troponin.

In case of troponin elevation in the screening setting, ICI therapy must be withhold (Brahmer et al., 2018). In order to diagnose myocarditis or other CV diseases as soon as possible—and to restart ICI therapy if myocarditis is not confirmed (Spallarossa et al., 2019; Palaskas et al., 2020)—it is mandatory for troponins to be carefully interpreted in the context of a comprehensive clinical evaluation. Patients must be asked for symptoms such as chest pain, dizziness, palpitations, and dyspnea, and a 12-lead ECG should be immediately performed. Troponins (and CPK) should be re-checked within 24 h, also measuring natriuretic peptides. Transthoracic echocardiography should be performed within 24–48 h, unless clinical instability requires urgent execution **(** **Figure 1** **)**. Troponin leakage in itself is not specific and represents myocardial injury, which may be related to acute coronary syndromes (acute coronary artery plaque event), type 2 myocardial infarction (mismatch in oxygen demand/supply due to, among other causes, anemia or tachy-arrhythmia with or without stable coronary lesions), myocarditis, cardiomyopathy, heart failure (Spallarossa et al., 2019; Thygesen et al., 2019). Patients with known CV disease, CV risk factors or in severe clinical conditions are more likely to show troponin abnormalities that might not directly depend on ICIs therapy. In contrast, patients with neither history of CV diseases nor experiencing stressors such as fever, anemia, oxygen desaturation, or tachycardia should raise suspicion, especially in case of persistent troponins elevation **(** **Figure 2** **)**.

These considerations, finally, highlight the potential benefit of a careful baseline cardiac evaluation. For instance, we noticed that during the screening phase with troponins in lung cancer patients receiving ICIs, troponins may be elevated in a significant proportion of patients. The entity of this increase is minimal in most cases, just above the upper limit of normal, in a pattern resembling that of chronic heart failure, in particular in patients with known cardiac disorders or with a severely frail state and progression of cancer (Sarocchi et al., 2018).

Importantly, is often necessary to repeat ECG, echocardiography, troponins, and physical examination, as a fundamental aspect is the dynamics of their changes. A slow and relatively small, but steady elevation of troponins is consistent with a diagnosis of myocarditis (Jaffe, 2011; Mahajan and Jarolim, 2011; Eggers and Lindahl, 2017). In the case of minimal troponin abnormalities, ECG and echocardiography may be normal (Caforio et al., 2013), and should be repeated after few days if clinical suspicion and troponins elevation persist. If troponins remains high, with no other identifiable causes, cardiac magnetic resonance may be useful (Caforio et al., 2013; Bonaca et al., 2019). Clearly, these considerations are not valid if the patient is hemodynamically unstable: this scenario requires immediate admission to an intensive cardiac care unit and an aggressive diagnostic and therapeutic work-up.

When a diagnosis of myocarditis is reached or the suspicion of myocarditis is strong, the patient should be admitted to a cardiology ward. If troponin testing shows normalization of values, and CPK values had always remained normal, without clinical, ECG, or echocardiographic alterations, ICIs may be restarted since a definite diagnosis of myocardial irAE cannot be achieved (Puzanov et al., 2017). Similarly, if troponin positivity is deemed to be related to cardiac disorders different from myocarditis, it may be reasonable to consider, on case-by-case basis, a re-challenge of ICIs. The specific cardiac disorder causing troponin leakage is obviously important; in some instances, such as if ACS is suspected, hospitalization may be reasonable.

Finally, when a definite diagnosis of myocarditis is reached or cannot be excluded, ICIs are not to be re-started, even if clinical, ECG, and echocardiographic findings are within normal range (Puzanov et al., 2017; Brahmer et al., 2018).

#### Step 4: Diagnosis of ICI-Related Myocarditis

A diagnostic workup for ICI-related myocarditis starts either in asymptomatic subjects with positive results of troponins screening, as previously discussed, or in symptomatic patients (**Figure 1**).

ICI-related myocarditis (and myocarditis in general) may have a various degree of presentation, from indolent and subclinical to life-threatening. Symptoms include dyspnea, chest pain, fatigue, palpitations, dizziness, and syncope (Caforio et al., 2013), resulting from left and/or right ventricular dysfunction, pericardial involvement with or without pericardial effusion, and atrial and/or ventricular brady- or tachy-arrhythmias. The golden rule is to never underestimate these symptoms in the context of therapies with ICIs, considering myocarditis as a real possibility, as it may rapidly escalate to cardiogenic shock.

Our institutional protocol provides for all symptomatic patients to be withheld ICI therapy and to undergo emergency cardiology consultation with the following diagnostic work-up:

1. Careful symptom interpretation and physical examination;
2. Serial troponins measurement;
3. Twelve-lead ECG;
4. Echocardiogram;
5. Natriuretic peptides;
6. CPK;
7. Chest X-ray.

If myocarditis or any new CV disorder are ruled out, the patient is not hospitalized, but re-examined in the short term for possible ICIs resumption. Otherwise, patients are admitted to the cardiology ward or the cardiac intensive care unit, according to the severity of clinical condition.

When the patient life is deemed safe by appropriate monitoring or hospital admission, cardiac magnetic resonance and endo-myocardial biopsy is to be considered as per myocarditis clinical guidelines (Caforio et al., 2013).

#### Step 5: Management of ICI-Related Myocarditis

According to current recommendations, the pivotal aspect in the management of myocarditis resides in the evaluation of the haemodynamic status (Caforio et al., 2013). Of note, despite ICI-related myocarditis may have a benign presentation and an uncomplicated course, yet a significant proportion of patients may present with haemodynamic and/or arrhythmic instability (Veronese and Ammirati, 2019). In such cases, it is important that patients are admitted to intensive care units with capability for haemodynamic monitoring, cardiac catheterization, and use of cardio-pulmonary assist devices—since conventional pharmacological therapy may not suffice (Caforio et al., 2013).

Published guidelines for myocarditis suggest immunosuppressive therapy only once a viral aetiology has been ruled out with endo-myocardial biopsy, or when myocarditis is associated with known (non-cardiac) autoimmune disorders (Caforio et al., 2013). This latter consideration holds true for ICI-related myocarditis, in which treatment is mostly based on the use of glucocorticoids (Puzanov et al., 2017; Brahmer et al., 2018). The American Society of Clinical Oncology and the Society for Immunotherapy of Cancer guidelines recommend to start with 1–2 mg/kg of oral or intravenous steroids, to be tapered in the following 4 to 6 weeks (Palaskas et al., 2020). Response is usually evaluated clinically and by measuring troponins levels. If troponins rise again during tapering, corticosteroids dosage should be increased again and attempts at tapering should be postponed (Palaskas et al., 2020). Guidelines recommend other immune-modulator therapies only in cases without immediate response to steroids. These may include mycophenolate, infliximab, anti-thymocyte globulins, alemtuzumab, and abatacept (Puzanov et al., 2017; Brahmer et al., 2018; Palaskas et al., 2020). These last two drugs, in particular, should be used with caution, since few cases have been reported (Palaskas et al., 2020). Moreover, high-dose infliximab is contraindicated in left ventricular dysfunction (Brahmer et al., 2018).

### Strengths and Weakness of Our Approach (New Section)

CV irAEs due to ICIs have received great attention in recent times. Several aspects of our approach, in particular in terms of screening strategies and therapeutic concepts, are in keeping with current guidelines recommendations (Puzanov et al., 2017; Brahmer et al., 2018; Hu et al., 2019; Palaskas et al., 2020) and recent dedicated literature (Michel et al., 2019; Tajiri and Ieda, 2019). However, the way we monitor CV irAEs and specifically ICI-related myocarditis focus on teamwork and organization. The approach we herein propose requires a tight co-operation between oncologists and cardiologists, involves prompt execution of diagnostic tests in case of justified suspicions, and is driven by recognition of abnormalities in biomarkers (troponin and CPK). A baseline cardiac evaluation is crucial to interpret the data. We acknowledge that our approach may be perceived as too demanding by clinicians and caregivers. Nevertheless, it is again worth to underline that in the absence of a structured programme of cardio-oncologic vigilance, the great majority of myocarditis events may be overlooked, or misdiagnosed. A recent meta-analysis of randomized clinical trials found that without a proactive surveillance it is not possible to detect significant cardiotoxicity associated with use of anti-PD-1/PD-L1 immunotherapy (Rahouma et al., 2019). The clinical trial reporting system, yet burdened by sometimes-misleading definitions, also strongly relies on investigator judgment for reporting adverse events. In clinical trial as well as in the real world, symptoms of myocarditis could be mistakenly attributed to other cardiac conditions, non-cardiac adverse effects of cancer therapies, or even to manifestations of the malignancy itself. Our integrated approach may, in part, overcome the pitfalls of current pharmacovigilance processes.

## Conclusions

Cardiologists should be aware of CV irAEs due to ICIs, since these effective anticancer drugs will be increasingly used. Different type of CV irAEs may occur, and their diagnosis and management should follow cardiology practice and guidelines, with the notable exception of myocarditis.

ICI-related myocarditis represents a clinical challenge both for diagnosis and management, and may require a peculiar immunosuppressive treatment. Moreover, there is still a paucity of data regarding clinical course of ICI-related myocarditis, in particular for the less dramatic cases. Nevertheless, ICI-related myocarditis may be severe or fatal, with absence or only a short period of alerting symptoms. Clinicians, however, may benefit from the fact that these events tend to occur within few weeks after ICIs initiation, thus allowing a troponins-based screening.

Clinical experience shows that a baseline CV evaluation (prior to initiation of ICIs therapy) is of paramount importance for a correct interpretation of both troponin elevation and unexpected clinical presentations. Timely and accurate diagnostic strategies are required to promptly confirm or rule out myocarditis at an early stage and to allow initiation of immunosuppressive therapies. However, uncertainty remains regarding several critical aspects of ICI-related myocarditis, from the screening phase to the clinical presentation, and further studies are needed to better understand this relevant condition.

## Author Contributions

PS contributed conception and design of the study. PS, GT, and MS wrote the first draft of the manuscript. PA, MT, IP, and EA wrote sections of the manuscript. All authors contributed to the article and approved the submitted version.

## Conflict of Interest

The authors declare that the research was conducted in the absence of any commercial or financial relationships that could be construed as a potential conflict of interest.
